# Psychometric properties of the Depression Stigma Scale in the Portuguese population and its association with gender and depressive symptomatology

**DOI:** 10.1186/s12955-022-01945-7

**Published:** 2022-03-05

**Authors:** Virgínia Conceição, Inês Rothes, Milton Severo, Kathleen Griffiths, Ulrich Hegerl, Ricardo Gusmão

**Affiliations:** 1 EPIUnit - Institute of Public Health, University of Porto, Porto, Portugal; 2 Laboratory for Integrative and Translational Research in Population Health (ITR), Porto, Portugal; 3 Faculty of Psychology and Education Science, University of Porto, Porto, Portugal; 4 Center for Psychology at, University of Porto, Porto, Portugal; 5 Department of Public Health and Forensic Sciences, and Medical Education, Faculty of Medicine, University of Porto, Porto, Portugal; 6 Research School of Psychology, College of Health and Medicine, The Australian National University, Canberra, Australia; 7 Department of Psychiatry, Psychosomatics, and Psychotherapy, Goethe-Universität Frankfurt, Frankfurt, Germany

**Keywords:** Personal stigma, Perceived stigma, Stigma scale, Depression stigma Portuguese population

## Abstract

**Background:**

Stigma is one of the most significant constraints on people living with depression. There is a lack of validated scales in Portugal to measure depression stigma; therefore, the Depression Stigma Scale (DSS) is essential to the depression stigma research in Portugal.

**Methods:**

We developed the adaptation process with the ITC Guidelines for Translation and Adapting Tests taken into consideration. We collected the sample as part of the OSPI program—Optimizing suicide prevention programs and their implementation in Europe, specifically within the application in Portugal, and included 1693 participants. Floor-ceiling effects and response ranges were analyzed, and we calculated Cronbach alphas, and Confirmatory Analysis. Validity evidence was tested with two well-documented hypotheses, using data on gender and depression symptoms.

**Results:**

The sample was well comparable with the general Portuguese population, indicating its representativeness. We identified a three-factor structure in each subscale (personal and perceived stigma): weak-not-sick, discrimination, and dangerous/unpredictable, with good model fit results. The Cronbach's alphas were satisfactory, and validity was confirmed.

**Conclusions:**

This study established the validity and demonstrated good psychometric properties of the DSS in the Portuguese population. The validation of the DSS can be beneficial in exploring stigma predictors and evaluating the effectiveness of stigma reduction interventions.

**Supplementary Information:**

The online version contains supplementary material available at 10.1186/s12955-022-01945-7.

## Introduction

The stigma associated with mental illnesses has been the subject of extensive study in recent years [1–4]. Two relevant concepts of stigma are social and self-stigma. Social Stigma is characterized by harmful attitudes and discriminatory behaviour towards people with mental illness [5]. Self-stigma results from the internalization of stigmatizing beliefs by the person living with mental illness [5], which often causes deep feelings of shame and guilt and may compromise the help-seeking and treatment process [6].

Recent research has focused on two types of social stigma: personal stigma and perceived stigma [7]. Personal stigma refers to one's beliefs about depression, and perceived stigma refers to one's beliefs about the attitudes of others.

There is a consensus on the adverse effects of mental illness stigma, such as reinforcing some pathological symptoms [8] and constraints on professional integration and access to mental health care [6, 9]. These effects have driven the stigma research from the general concept of mental illness to the specific stigma attributed to a particular diagnosis.

Stigma is one of the most significant constraints on people living with depression, with the community and stigmatized institutional responses similar to those experienced by people with psychosis or chronic mental illness [10]. Experience of discrimination associated with a depressive condition occurred with 79% of research participants, and between 20 and 37% have compromised their actions because of anticipated discrimination [8]. Embarrassment and discrimination experiences afflict 14.8% of people with common mental disorders in Europe, more common among individuals with lower education and those married [11].

In 2015, the estimated prevalence of depression worldwide was 4.4% [12]. In contrast, in Portugal, the prevalence in the same year was estimated at 5.7% and is responsible for 8.5% of the total Years Lived with Disability [12], raising the importance of depression stigma research.

Even though research about depression and stigma association is increasing, it is essential to widen the range of socio-cultural contexts of the studies [13], and the adoption of an instrument used in a variety of countries would allow overcoming difficulties related to the methodological discrepancies between existing studies [14].

Currently, in Portugal, there are a few validated instruments to evaluate the stigma associated with mental illness, such as The Internalized Stigma of Mental Illness [15], the Attribution Questionnaire-27 [16], the Attribution Questionnaire-9 [17], the Mental Illness Beliefs Inventory [18], and the Opinions about Mental Illness Scale [19]. All the mentioned questionnaires evaluate beliefs and stigmatized attitudes towards mental illness or people living with mental illness, with non-specification by diagnosis. Currently, there is a lack of instruments evaluating the stigma associated with depression in Portugal.

Although data about depression stigma in Portugal was published under the studies resulting from the Optimizing suicide prevention programs and their implementation in Europe (OSPI) research program [20, 21], and the psychometric properties of the instrument used were explored at the time, the validation of the Depression Stigma Scale [22] was not yet published and is an essential step to the depression stigma research in Portugal.

The Depression Stigma Scale was developed by the Centre for Mental Health Research at the Australian National University to measure the stigma associated with depression [23]. It has two subscales that measure two different types of stigma: personal and perceived. The Personal Stigma Subscale measures stigma in the respondent's attitudes towards depression by indicating how strongly they agree with nine statements about depression. The Perceived Stigma Subscale measures the respondent's perception about the attitudes of others towards depression by asking them to indicate what they think most other people believe about the same nine statements. Measurement of responses to each item are on a five-point scale (ranging from zero 'strongly disagree' to four 'strongly agree'). Higher scores indicate higher levels of depression stigma.

The Depression Stigma Scale has been described as an excellent instrument to evaluate the way someone interacts with another person with depression, defining stigma as a negative attitude towards a person, which leads to negative action or discrimination [23] and has been used in recent studies in a wide range of cultures [2, 24–26]. Thus, the detailed study of its psychometric properties is of value to understanding and researching depression stigma and studying the effects of anti-stigma campaigns and the development of effective stigma-reduction interventions.

With this study, we intend to test the following hypothesis:

- In the Portuguese population, the Depression Stigma Scale maintains the two-subscales structure, corresponding to the Personal Depression Stigma and Perceived Depression Stigma, respectively;
- The scale presents good psychometric properties for the Portuguese population;
- Each subscale presents a three-factor solution;
- The scale validity is confirmed.

## Methods

We took the ITC Test Translation and Adaptation Guidelines [27] into consideration in the scale adaptation process and the second edition [28] in the validation process.

We obtained permission from the original developer of the scale that actively collaborated in the adaptation process, one of this work's authors. Furthermore, since the adaptation occurred during the OSPI program [20], a group of experts on cultural differences of the stigma construct was involved in the construct-item match, evaluating its suitability for the Portuguese language and evaluating potential cultural differences.

### Adaptation process

The translation of the Depression Stigma Scale (DSS) Portuguese version followed the double-translation and reconciliation procedure: two independent researchers, Portuguese natives, translated the questions from English to Portuguese, then the differences in translation were analyzed and conciliated by a third Portuguese native depression stigma expert. We then asked a group of native Portuguese to review the scale to ensure that the test instructions and item content had the intended meaning, with particular account for the stigma context in Portugal. This last group of researchers was both specialists in depression as well as in stigma.

Since the DSS is a five-point Likert scale, with answers ranging in agreement, and this is a widespread method of scale construction in Portugal, the item format was considered suitable.

The only localized adaptation to the original version had to do with the absence of gender-neutral language in Portugal, so, in some items, we adapted the question for both male and female forms of the words.

### Participants

The sample collection for this study was part of the OSPI program – Optimizing suicide prevention programs and their implementation in Europe, specifically within the application in Portugal [11, 29], and the current study assumed a cross-sectional study design.

The adopted methodology for sample collection was discussed in-depth and approved by the project consortium.

Since there are no listings of mobile phones in Portugal, we extracted a selection of participants from the cable telephone network listed numbers, using the random digit dialling method to numbers belonging to Almada and Amadora municipalities. Trained interviewers conducted telephone contacts, and fieldwork was performed at the end of the day and on weekends to achieve data representativeness regarding gender and age.

Almada and Amadora combined have above 350,000 inhabitants, making them two of the most populous counties in Portugal. Due to its high urban population density and the socio-economic diversity of its inhabitants, these municipalities were considered representative of the Portuguese population [20, 21].

The participation rate was 46%, considering the response rate has been decreasing in the past decades [30], and the mean response rate in 2012 for telephone surveys was 30.2%, the sample consent bias was considered minimal. The increasing number of surveys, information requests, and the rise of privacy-related issues are the main reasons for the decay of the participation rate [30].

Of the 2009 participants in the OSPI research program [20, 21], we excluded 316 participants from the current study due to their exposure to the OSPI intervention aimed to promote literacy on depression and, consequently, help-seeking behaviour. The collection of data occurred between 2009 and 2010.

### Instruments

We asked participants to answer a questionnaire containing sociodemographic queries concerning the respondent's sex, age, professional occupation, and the complete Depression Stigma Scale, as well as the Portuguese version of the Mental Health Inventory 5 (MHI-5) [31, 32] to screen for depressive symptoms.

The MHI-5 is a brief version of the 38-item MHI developed in 1983 [33]. The MHI-5 was developed for use with the general population, and it includes items on psychological well-being. The five 6-point Likert items access psychological well-being (2 items) and the absence of psychological distress (3 items). The Portuguese version shows good psychometric properties, with a reliability above 0.80, moderate correlation with comparison measures, and shows the same pattern as that of the longer form, and the correlation between the long and short form is r = 0.95 [32].

### Data analysis

In addition to computing a continuous score for each DSS, we transformed the DSS score into percentages, following the original scale [22], with higher percentages indicating greater stigma levels. The same procedure was carried out for the MHI-5 scale, transforming the scores in the 0–100 range; higher scores meant better mental health than lower scores.

To study the psychometric properties of the DSS, we analyzed (a) floor-ceiling effects and response ranges; (b) as a reliability estimation, we calculated Cronbach alphas in order to maintain reliability comparability with the original scale; and (c) conducted a principal component analysis and tested the model Fit using Confirmatory Factor Analysis.

Since most recent validations showed different a three-factor solution in each subscale [34, 35], we conducted Confirmatory Factor Analysis to analyse the structure of the scale in the Portuguese population. We named the first and last factors after Zhu and colleagues' designation [35]: weak-not-seek in the first factor (items 1, 2, 3 in the personal subscale and 10, 11, 12 in the perceived subscale) and discrimination in the third factor (items 7, 8, 9 in the personal subscale and 16, 17, 18 in the perceived subscale). The second factor was labelled Dangerous/ unpredictable, following Boerema and colleague's designation [34], corresponding to items 4, 5, 6 in the personal subscale and 13, 14, and 15 in the perceived subscale.

Descriptive statistics of the DSS Personal and Perceived items were estimated, along with the corrected item-test correlation.

In the Confirmatory Factor Analysis, we tested the model fit of the extracted structure of each subscale using the Comparative Fit Index (CFI), Tucker- Lewis Index (TLI), Root Mean Square Residual (SRMR), and Root Mean Square Error of Approximation (RMSEA). The criteria for an acceptable or good model fit were CFI and TLI > 0.95, SRMR < 0.06, and RMSEA < 0.08 were considered an acceptable model fit [36].

We based validity evidence on the test of two of the better-documented hypotheses [7, 34, 37]: (1) women present lower scores of personal stigma and higher perceived stigma than men, and (2) depressive symptomatology is associated with higher personal and perceived stigma. We calculated means and standard deviations for continuous variables, student t-tests to examine the differences between sexes, and separate univariate linear regression analyses to assess the effects of symptomatology and other factors on each depression stigma subscale, as well as Pearson correlations.

IBM SPSS Statistics, Version 24.0 software package was used to conduct statistical analysis, and RStudio was used to conduct the Confirmatory Analysis.

## Results

### Sample description

Our study included 1693 participants. We describe the sociodemographic characteristics of the sample in Table 1. National data estimates a mean age in the Portuguese population of 45.2, with a gender distribution of 52.2% women and 47.8% men, so our data is well comparable with the national population.

**Table 1:** Sample sociodemographic characteristics

Age	Mean (SD)	
	47.23 (18.17)	
Sex n (%)	Men	786 (46.4%)
	Female	907 (53.6%)
Marital status N (%)	Married	896 (52.9%)
	Single	562 (33.2%)
	Widowed	120 (7.1%)
	Divorced	115 (6.8%)

As we can observe in Table 1, most of the individuals in our sample were married, followed by single individuals, widowed, and divorced. Thus, the marital status of the Portuguese population follows a similar distribution: 54.8% are married, 30.1% are single, 8.5% are widows, and 6.6% are divorced.

In addition, we found 8.6% of unemployment in our sample, whereas in 2009, the unemployment rate in Portugal was 9.4%.

### Depression Stigma Scale psychometric characteristics

In the full scale, we obtained a Determinant of 0.22. The KMO (Kaiser–Meyer–Olkin) test value is sufficiently close to 1 (KMO = 0.78), which indicates that the correlation patterns are relatively compact, so factorial analysis should produce factors other than reliability. The Bartlett test of sphericity shows that the correlation matrix is significantly different from the identity matrix (*p* < 0.001), confirming that it is appropriate to perform the factor analysis. There were no missing values, and we used all response options in all items. Besides, there were no evident inconsistencies in the frequency of responses, and no ceiling or floor effects were detected (Tables 2 and 3).

**Table 2:** Means, standard deviations, Kolmogorov–Smirnov value, and Kurtosis for every scale item

Item	M	SD	Sk	Ku
1	2.88	1.18	0.044	− 1.272
2	2.57	1.20	0.446	− 1.042
3	2.35	1.11	0.697	− 0.534
4	2.81	1.07	0.114	− 1.062
5	2.33	1.08	0.726	− 0.443
6	3.51	0.91	− 0.829	− 0.122
7	2.25	1.01	0.846	0.029
8	2.11	0.88	1.044	1.064
9	2.46	1.11	0.75	− 0.327
10	3.25	1.06	− 0.388	− 1.138
11	3.22	1.05	− 0.376	− 1.179
12	3.13	1.05	− 0.247	− 1.249
13	2.92	1.00	0.117	− 1.274
14	3.13	1.07	− 0.195	− 1.252
15	3.52	0.86	− 0.768	− 0.334
16	3.45	0.95	− 0.596	− 0.766
17	3.30	0.99	− 0.315	− 1.069
18	3.24	1.02	− 0.197	− 1.143

**Table 3:** Variance accounted for each factor

	Factor	R^2^
Personal subscale	Weak-not-seek	0.52
	Dangerous/unpredictable	0.78
	Discrimination	0.56
Perceived subscale	Weak-not-seek	0.53
	Dangerous/unpredictable	0.73
	Discrimination	0.52

In Table 4, we can observe the Portuguese version of the items. The scale presented internal consistency results with a Cronbach's Alpha of the full scale of 0.75, 0.71 in the Personal Depression Stigma Subscale, and 0.75 in the Perceived subscale. The alpha value did not increased with the removal of any of the items, as we can observe in Table 4.

**Table 4:** Measures of sampling adequacy in the full Depression Stigma Scale, Parameter estimates in Personal and Perceived subscales, and internal consistency of personal and perceived depression stigma subscales

Item		Measure of sampling adequacy		Parameter estimates (Std. lv)	Cronbach's alpha	Item-total correlation
		1	2			
1	As pessoas com depressão poderiam sair dela se quisessem	0.70		0.456	0.686	0.480
2	A depressão é um sinal de fraqueza	0.73		0.910	0.731	0.523
3	A depressão não é uma verdadeira doença médica	0.75		0.626	0.659	0.402
4	As pessoas com depressão são perigosas	0.77		0.374	0.667	0.465
5	É melhor evitar pessoas com depressão para não se tornar deprimido/a também	0.77		0.646	0.756	0.420
6	As pessoas com depressão são imprevisíveis	0.72		0.332	0.680	0.496
7	Se eu tivesse uma depressão não diria a ninguém	0.75		0.442	0.696	0.408
8	Não empregaria alguém se soubesse que ele/ela tivesse tido uma depressão	0.70		0.616	0.660	0.417
9	Não votaria num político se soubesse que ele tivesse tido uma depressão	0.67		0.705	0.761	0.397
10	A maioria das pessoas acha que as pessoas com depressão poderiam sair dela se quisessem		0.72	0.558	0.748	0.414
11	A maioria das pessoas acha que a depressão é um sinal de fraqueza		0.74	0.794	0.718	0.490
12	A maioria das pessoas acha que a depressão não é uma verdadeira doença médica		0.77	0.625	0.734	0.398
13	A maioria das pessoas acha que as pessoas com depressão são perigosas		0.81	0.474	0.723	0.464
14	A maioria das pessoas acha que é melhor evitar pessoas com depressão para não se tornar deprimido/a também		0.81	0.810	0.708	0.548
15	A maioria das pessoas acha que as pessoas com depressão são imprevisíveis		0.81	0.375	0.737	0.377
16	A maioria das pessoas não diria se tivessem depressão		0.81	0.565	0.728	0.437
17	A maioria das pessoas não empregaria alguém que soubesse ter tido uma depressão		0.74	0.736	0.720	0.485
18	A maioria das pessoas não votaria num político se soubesse que ele tinha tido uma depressão		0.68	0.578	0.741	0.455

**Fig. 1:**
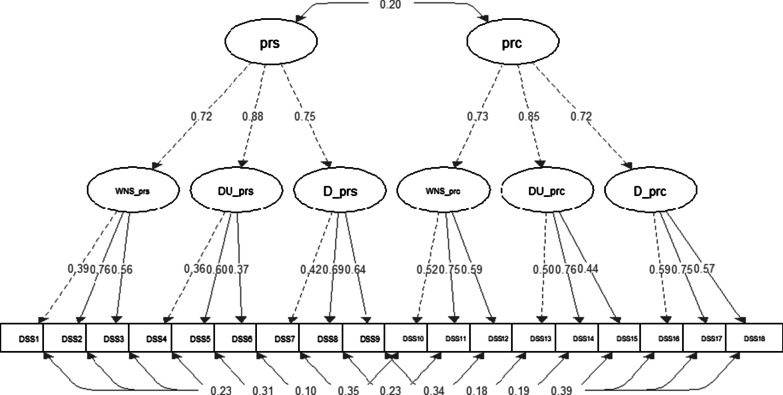
Final CFA model for de Depression Stigma Scale. prs = Personal Depression Stigma Subscale, prc = Perceived Depression Stigma Subscale, WNS_prs = Weak-not-seek in the personal subscale, DU_prs = Dangerous/unpredictable in the personal subscale, D_prs = Discrimination in the personal subscale, WNS_prc = Weak-not-seek in the perceived subscale, DU_prc = Dangerous/unpredictable in the perceived subscale, D_prc = Discrimination in the perceived subscale

The CFA analysis for the three-factor solution in each subscale showed a good model fit: χ^2^_(df)_ = 602_(123)_, *p* < 0.001 CFI = 0.92, TLI = 0.90, SRMR = 0.05, and RMSEA = 0.04. The model also presented good standardized estimates as we can observe in Fig. 1.

Each of the three factors within the subscales accounted for more than 50% of the variance, as we can see in Table 3.

The covariance for each corresponding item in both scales was always positive, ranging from 0.10 to 0.39, as shown in Fig. 1.

### Validity evidence

In the personal stigma subscale, women presented a statistically lower mean score (M = 38.29, SD = 13.98) than men (M = 41.02, SD = 13.95), t_(1691)_ = 4.00, *p* < 0.001, CI = 3.24, 5.03, with a good size effect (*d* = 0.74). On the other hand, women obtained higher mean scores on the perceived subscale (M = 56.53, SD = 14.83) than men (M = 55.38, SD = 13.89). However, the difference was not significant (t_(1691)_ =  − 1.64, *p* = 0.10, CI =  − 2.21, 1.98).

MHI-5 scores showed a negative correlation with the personal depression stigma (r =  − 0.07, *p* < 0.01); still, the correlation with the perceived depression stigma subscale was not significant (r = 0.02, *p* = 0.30).

We detected significant effects on personal stigma from gender, age, and depressive symptomatology in the regression analysis (full regression results can be found in Additional file 1: Table S1 "Effects of gender, age, and MHI-5 on personal depression stigma scores"). Both age and being a man have positive effects on personal stigma, increasing their score. On the other hand, better MHI-5 scores, translating into better mental health, had a negative effect on personal depression stigma, decreasing the score.

On the perceived depression stigma, gender and age presented the opposite effect compared to the personal stigma: age and being a man decreased the perceived depression stigma score, (as shown in Additional file 1: Table S2 "Effects of gender, age, and MHI-5 on perceived depression stigma scores"). MHI-5 scores did not produce a significant effect on perceived stigma scores.

## Discussion

The present study is the first to examine the Depression Stigma Scale (DSS) psychometric properties in the Portuguese population.

Even though our sample presented a mean age 5 years older than the mean age of the Portuguese population, we can consider our sample representative due to the similarities in other sociodemographic characteristics, such as gender distribution, occupation, and marital status.

Overall, the Portuguese version of the DSS showed good psychometric properties, suggesting that it was an appropriate instrument for future studies in the Portuguese population.

We calculated the internal consistency for the nine subscale items; therefore, we can compare our results with other validations in other countries' general populations.

Although the perceived subscale internal consistency was lower than that obtained in its original form (α = 0.88) [22], and in the Dutch version (α = 0.82) [34], it was nevertheless satisfactory. Internal consistency for the personal subscale was similar to that reported in previous studies.

We confirmed each subscale as a particular dimension from the full scale, and all the items in each subscale presented good confirmatory loadings. Additionally, and in similarity with the structure identified by Boerema and colleagues [34], the scree plot of each subscale indicated a three-factor solution: *weak-not-sick* (items 1, 2, and 3 from the personal subscale and 10, 11, and 12 in the perceived subscale), *dangerous/unpredictable* (items 4, 5, 6 and 13, 14 and 15 form the personal and perceived subscale respectively) and lastly, *discrimination* (items 7, 8 and 9 in the personal subscale and 16, 17 and 18 in the perceived subscale). This structure showed a good fit indices in the confirmatory factor analysis, an essential finding for better understanding the scale structure in the Portuguese population since the scale structure seems to be influenced by cultural factors, ranging from a one-factor structure [38] to a three-factor [34].

In order to produce evidence of validity, we tested two well-documented hypotheses [7, 34, 37]. The first one, widely observed in previous literature, stated that we expected to see women with lower personal stigma and higher perceived stigma than men. When analyzing mean differences between genders, the difference between scores in the personal scale was clear: women showed lower personal stigma than men in agreement with the previous literature [7, 22, 34, 37]; however, we observed no difference in the perceived depression stigma subscale. Thus, interpretations of differences in perceived stigma have not been as consensual in the literature as those observed in personal stigma. On the one hand, we can find research that supports that women present a higher perceived stigma than men [37]; on the other hand, we find literature supporting the absence of differences [39]. We found no statistically significant difference between genders in the perceived scale; however, when we performed the regression, gender had a significant effect on perceived depression stigma scores, which can indicate that there are, however subtle, gender differences conditioned by other variables, such as previous experience with depression and help-seeking intentions.

The second hypothesis led us to expect more significant personal and perceived stigma in the population with the higher depressive symptoms. While, in our sample, we confirmed the hypotheses for personal stigma, in the perceived depression stigma, depressive symptoms did not show significant effects. Therefore, we can hypothesize that perceived depression stigma can be more sensitive to other variables such as the proximity of the people participants think when evaluating stigma around them [40] and previous help-seeking experiences and close ones living with depression [5, 41].

One limitation of this study is related to the data collection because interviews were carried out by phone, probably delivering some desirable social answers. Another limitation is the absence of convergent validity of the scale, and future research is needed to examine the convergent validity in the Portuguese population.

The relationship between personal and perceived depression stigma should also be further studied in future research since the subscales presented a weak correlation.

Lastly, future studies may also consider the evaluation of the psychometric properties of the scale in a more rural setting of the country since even though the sample of the study is well comparable with the general Portuguese population, rural residents may be underrepresented.

## Conclusions

The scale presented good psychometric properties in the Portuguese population, and its validity was confirmed. Considering the well-recognized adverse effects of stigma, the development of many initiatives aimed to reduce depression stigma in the populations, the existence of a validated scale can be crucial in evaluating the effectiveness of the interventions, as well as adjust and adapt future interventions according to the best depression stigma reduction tools. Also, access to a validated scale allows us to explore better the depression stigma predictors and their effects on help-seeking and mental health promotion behaviours.

## Supplementary Information

**Additional file 1.** Effects of gender, age, and MHI-5 on personal depression stigma scores.

## Data Availability

The datasets analyzed during the current study are available from the corresponding author on reasonable request.

## References

[1] Shahwan S, Lau JH, Goh CMJ, Ong WJ, Tan GTH, Kwok KW (2020). The potential impact of an anti-stigma intervention on mental health help-seeking attitudes among university students. BMC Psychiatry.

[2] Yang F, Yang BX, Stone TE, Wang XQ, Zhou Y, Zhang J (2020). Stigma towards depression in a community-based sample in China. Compr Psychiatry.

[3] Kosyluk K, Marshall J, Conner K, Macias DR, Macias S, Michelle Beekman B (2021). Challenging the stigma of mental illness through creative storytelling: a randomized controlled trial of this is my brave. Commun Ment Health J.

[4] Walsh DAB, Foster JLH (2021). A call to action. A critical review of mental health related anti-stigma campaigns. Front. Public Health.

[5] Corrigan PW (2006). Mental health stigma as social attribution: Implications for research methods and attitude change. Clin Psychol Sci Pract.

[6] Rusch N, Angermeyer MC, Corrigan PW (2005). Mental illness stigma: concepts, consequences, and initiatives to reduce stigma. Eur Psychiatry.

[7] Griffiths KM, Christensen H, Jorm AF (2008). Predictors of depression stigma. BMC Psychiatry.

[8] Lasalvia A, Zoppei S, Van Bortel T, Bonetto C, Cristofalo D, Wahlbeck K (2013). Global pattern of experienced and anticipated discrimination reported by people with major depressive disorder: a cross-sectional survey. Lancet.

[9] Kashihara J. Examination of stigmatizing beliefs about depression and stigma-reduction effects of education by using implicit measures. (0033–2941 (Print)) 10.2466/15.PR0.116k20w9 25748084

[10] McNair BG, Highet NJ, Hickie IB, Davenport TA (2002). Exploring the perspectives of people whose lives have been affected by depression. Med J Aust.

[11] Alonso J, Buron A, Rojas-Farreras S, de Graaf R, Haro JM, de Girolamo G (2009). Perceived stigma among individuals with common mental disorders. J Affect Disord.

[12] World Health Organization. Depression and Other Common Mental Disorders: Global Health Estimates, Geneva; 2017.

[13] Martínez V, Crockett MA, Jiménez-Molina Á, Espinosa-Duque HD, Barrientos E, Ordóñez-Carrasco JL (2020). Stigmatizing beliefs and attitudes to depression in adolescent School Students in Chile and Colombia. Front Psychol..

[14] Kaushik A, Kostaki E, Kyriakopoulos M (2016). The stigma of mental illness in children and adolescents: a systematic review. Psychiatry Res.

[15] Oliveira SE, Esteves FG, Pereira EG, Carvalho M, Boyd JE (2015). The internalized stigma of mental illness: cross-cultural adaptation and psychometric properties of the Portuguese version of the ISMI scale. Commun. Ment Health J.

[16] Sousa S, Marques A, Rosário C, Queirós C (2012). Stigmatizing attitudes in relatives of people with schizophrenia: a study using the attribution questionnaire AQ-27. Trends Psychiatry Psychother.

[17] Oliveira ARF, Azevedo SM. Estigma na doença mental: estudo observacional. Revista Portuguesa de Medicina Geral e Familiar; v 30, n 4 (2014): Revista Portuguesa de Medicina Geral e Familiar. 2014.

[18] Loureiro L, Dias C, Ferreira P (2006). Um Inventário de Crenças acerca da Doença Mental. Revista de Investigação em Enfermagem.

[19] Oliveira S (2005). A loucura no outro: um contributo para o estudo do impacto da loucura no profissional de Saúde Mental.

[20] Hegerl U, Wittenburg L, Arensman E, Van Audenhove C, Coyne JC, McDaid D (2009). Optimizing suicide prevention programs and their implementation in Europe (OSPI Europe): an evidence-based multi-level approach. BMC Public Health.

[21] Coppens E, Van Audenhove C, Scheerder G, Arensman E, Coffey C, Costa S (2013). Public attitudes toward depression and help-seeking in four European countries baseline survey prior to the OSPI-Europe intervention. J Affect Disord.

[22] Griffiths KM, Christensen H, Jorm AF, Evans K, Groves C (2004). Effect of web-based depression literacy and cognitive-behavioural therapy interventions on stigmatising attitudes to depression: randomised controlled trial. Br J Psychiatry.

[23] Fusar-Poli P, de Pablo GS, De Micheli A, Nieman DH, Correll CU, Kessing LV (2020). What is good mental health? A scoping review. Eur Neuropsychopharmacol..

[24] Yuan Q, Seow E, Abdin E, Chua BY, Ong HL, Samari E (2018). Direct and moderating effects of personality on stigma towards mental illness. BMC Psychiatry.

[25] Singh S, Zaki RA, Farid NDN (2019). A systematic review of depression literacy: knowledge, help-seeking and stigmatising attitudes among adolescents. J Adolesc.

[26] Hanlon HR, Swords L (2020). Adolescent endorsement of the “weak-not-sick” stereotype for generalised anxiety disorder: associations with prejudice, discrimination, and help-giving intentions toward peers. Int J Environ Res Public Health.

[27] Hambleton RK (2001). The next generation of the ITC test translation and adaptation guidelines. Eur J Psychol Assess.

[28] ITC Guidelines for Translating and Adapting Tests (Second Edition). Int J Test 2017;18(2):101–34.

[29] Kohls E, Coppens E, Hug J, Wittevrongel E, Van Audenhove C, Koburger N (2017). Public attitudes toward depression and help-seeking: impact of the OSPI-Europe depression awareness campaign in four European regions. J Affect Disord.

[30] Beullens K, Loosveldt G, Vandenplas C, Stoop I. Response rates in the European Social Survey: Increasing, decreasing, or a matter of fieldwork efforts? Survey Methods: Insights from the Field. 2018.

[31] Berwick DM, Murphy JM, Goldman PA, Ware JE, Barsky AJ, Weinstein MC (1991). Performance of a five-item mental health screening test. Med Care.

[32] Pais-Ribeiro J (2001). Mental health inventory: Um estudo de adaptação à população portuguesa. Psicologia Saúde & Doenças.

[33] Rivera-Riquelme M, Piqueras JA, Cuijpers P. The Revised Mental Health Inventory-5 (MHI-5) as an ultra-brief screening measure of bidimensional mental health in children and adolescents. 10.1016/j.psychres.2019.02.045 30818147

[34] Boerema AM, Zoonen K, Cuijpers P, Holtmaat CJ, Mokkink LB, Griffiths KM (2016). Psychometric properties of the Dutch Depression Stigma Scale (DSS) and associations with personal and perceived stigma in a depressed and community sample. PLOS ONE.

[35] Zhu L, Yao J, Wu L, Wang J, Qiu M, Zhang C (2019). Psychometric properties of the Depression Stigma Scale (DSS) in Chinese cancer patients: a cross-sectional study. BMJ Open.

[36] Hu L-T, Bentler PM (1999). Cutoff criteria for fit indexes in covariance structure analysis: conventional criteria versus new alternatives. Struct Equ Model.

[37] Busby Grant J, Bruce CP, Batterham PJ (2016). Predictors of personal, perceived and self-stigma towards anxiety and depression. Epidemiol Psychiatr Sci.

[38] Dardas LA, Silva S, Noonan D, Simmons LA (2018). Studying depression among Arab adolescents: methodological considerations, challenges, and lessons learned from Jordan. Stigma and Health.

[39] Pyne JM, Kuc EJ, Schroeder PJ, Fortney JC, Edlund M, Sullivan G (2004). Relationship between perceived stigma and depression severity. J Nerv Ment Dis.

[40] Jennings KS, Cheung JH, Britt TW, Goguen KN, Jeffirs SM, Peasley AL (2015). How are perceived stigma, self-stigma, and self-reliance related to treatment-seeking? A three-path model. Psychiatr Rehabil J.

[41] Cornally N, McCarthy G (2011). Help-seeking behaviour: a concept analysis. Int J Nurs Pract.

