# Supplementary material for: Psychometric properties of the Depression Stigma Scale in the Portuguese population and its association with gender and depressive symptomatology
Source: Health Qual Life Outcomes. 2022 Mar 5;20:42. doi: 10.1186/s12955-022-01945-7 (PMC8898398; doi:10.1186/s12955-022-01945-7)
Supplement: Supplementary file 1 — Additional file 1. Effects of gender, age, and MHI-5 on personal depression stigma scores. [file 12955_2022_1945_MOESM1_ESM.docx]

**Supplementary file**

Table 1: Effects of gender, age, and MHI-5 on personal depression stigma scores

|  | **Β** | | | **95% CI** | **t** | | | **P** |
| --- | --- | --- | --- | --- | --- | --- | --- | --- |
| Woman | | Ref. |  | | |  |  | |
| Men | | **3.37** | **2.30, 4.65** | | | **26.87** | **<0.001** | |
| Age | | **0.23** | **0.18, 0.27** | | | **165.52** | **<0.001** | |
| MHI-5 | | **-0.10** | **-0.17, -0.02** | | | **6.41** | **<0.05** | |

β=beta regression coefficients, Ref.=Reference category

Significant results are shown in bold.

Table 2: Effects of gender, age, and MHI-5 on perceived depression stigma scores

|  | **Β** | | | **95% CI** | **t** | | | **P** |
| --- | --- | --- | --- | --- | --- | --- | --- | --- |
| Woman | | Ref. |  | | |  |  | |
| Men | | **-1.46** | **-2.82, -0.09** | | | **4.38** | **<0.05** | |
| Age | | **-0.01** | **-0.26, -0.08** | | | **30.04** | **<0.001** | |
| MHI-5 | | 0.03 | -0.05, 0.11 | | | 0.57 | 0.45 | |

β=beta regression coefficients, Ref.=Reference category

Significant results are shown in bold.
